# A Comparative Analysis of Thin-Layer Microwave and Microwave/Convective Dehydration of Chokeberry

**DOI:** 10.3390/foods12081651

**Published:** 2023-04-15

**Authors:** Marko Petković, Vladimir Filipović, Biljana Lončar, Jelena Filipović, Nemanja Miletić, Zoranka Malešević, Darko Jevremović

**Affiliations:** 1 Faculty of Agronomy, University of Kragujevac, Cara Dušana 34, 32102 Čačak, Serbia; n.m.miletic@kg.ac.rs; 2 Faculty of Technology, University of Novi Sad, Cara Lazara 1, 21000 Novi Sad, Serbia; vladaf@uns.ac.rs (V.F.); cbiljana@uns.ac.rs (B.L.); 3 Institute for Food Technology, University of Novi Sad, 21000 Novi Sad, Serbia; jelena.filipovic@fins.uns.ac.rs; 4 Faculty of Agriculture, University of East Sarajevo, 71126 Lukavica, Bosnia and Herzegovina; 5 Fruit Research Institute, 32102 Čačak, Serbia

**Keywords:** chokeberry, water holding capacity, thin-layer, semi-theoretical mathematical models, drying kinetics, sensory evaluation

## Abstract

Due to high water content, chokeberries (*Aronia melanocarpa* L.) are perishable. Therefore, energy-saving, combined drying technologies have been explored to improve the chokeberry drying. The combined microwave and the traditional convective drying method (MCD) have significantly enhanced the drying effectiveness, efficiency, and energy utilization rate and improved product quality. The MCD method, which implies the microwave power (MD) of 900 W for 9 s and the convective dehydration (CD) at 230 °C for 12 s, has the shortest dehydration time t (24 ± 2 min), has the maximum coefficient of diffusion (D_eff_ = 6.0768 × 10^−9^ ± 5.9815 × 10^−11^ m^2^ s^−1^), and represents the most energy effective for dehydration process (E_min_ = 0.382 ± 0.036 kWh). A higher water-holding capacity (WHC) characterized the chokeberries obtained by the MCD method compared to the regular microwave method (MD). The mildest MCD (15 s of MD on 900 W, 7 s of CD on 180 °C) could dehydrate chokeberries with the highest WHC (685.71 ± 40.86 g H_2_O g^−1^ d.m.) and the greatest evaluations for sensory attributes in terms of all properties. The results of this study provide the drying behavior of chokeberries that can help develop efficient drying methods and improve existing ones.

## 1. Introduction

Black chokeberry (*Aronia melanocarpa* L.) is a deciduous shrub species in the Rosaceae family. It is native to North America and commonly found in wetland areas and along streams and rivers. It is known for its dark, almost black fruit that is edible and high in antioxidants (e.g., polyphenols: procyanidins, anthocyanins, phenolic acids, and vitamins). The fruit can be used in making jams, jellies, syrups, and juices [1]. The plant is also valued for its ornamental value, with its white flowers in the spring and its red foliage in the fall. The growing interest in developing and optimizing the transformation process of berries with polyphenolic compounds from natural sources into powders or extracts is driven by the potential applications in the food, chemical, and pharmaceutical industries [2]. Chokeberry has a tart and bitter taste due to high levels of polyphenols such as proanthocyanidin tannins, phenolic acids, and bitter flavanones [3]. It is mostly consumed, after processing, in dried form for use in snacks, cereals, salads, and tea. The use of chokeberry in nutritional supplements has also gained popularity [4].

Berries such as chokeberry or aronia (*Aronia melanocarpa* L.) are popular due to their unique flavor and appearance, but they have high water content and easily deteriorate. Drying berries solves the storage and transportation issue while preserving the original taste, flavor, and nutrients. Common drying methods include hot air, vacuum, microwave, and freeze drying. The development of new, non-hot food processing technology is needed to optimize the drying process while reducing browning, degradation of functional components, and deterioration of flavor. Energy-saving and quality-upgrading combined drying technologies and physical field pretreatment technology can be used to improve the energy efficiency of berry drying.

The traditional convective dehydration process is a heat and mass transfer process between the drying medium (air, for example) and the material being dried (in this case, fresh berries). The heat energy is transferred from the drying medium to the surface of the material and then from the surface to the interior. The moisture then moves from the inside of the material to the surface in either liquid or gaseous form and diffuses into the air through the surface gas film. The driving forces behind this process are the temperature and humidity gradient between the drying medium and the material, which cause the internal water of the berries to vaporize and move outward. The ultimate goal of the drying process is achieved through the moisture gradient between the surface and interior of the material and the humidity gradient between the material surface and the drying medium [5]. Moisture diffusion in materials is divided into two stages: water in-diffusion and water out-diffusion. The out-diffusion stage refers to the evaporation of moisture from the material’s surface due to various factors such as air velocity, relative humidity, temperature, and surface area. In the in-diffusion stage, internal moisture transfers to the surface due to a difference in moisture content between the surface and interior. A balance between the two stages is essential in the drying process, as out-diffusion that is too fast can cause hard shells to form, and evaporation that is too slow can lead to material ripening and mildew [6,7]. The dehydration speed of the material can be divided into five stages: I—preheat period, II—constant rate period, III—first falling rate period, IV—second falling rate period, and V—equilibrium drying rate period [8]. In the constant drying rate period, increasing the temperature and reducing the humidity of the air can speed up the drying process as well as improve the material–air contact. During the falling drying rate period, the drying speed is mainly determined by the moisture diffusion rate within the material, so increasing the temperature of the material and improving its dispersion can help accelerate the drying process.

The microwave dehydration process works by transmitting electromagnetic waves through a medium; the heat produced by molecular vibration is then used to dehydrate berries. The organoleptic qualities of the products are comparable to those obtained by freeze drying, but the duration of microwave drying can be cut in half [9]. This method is typically used in industry to dry berries for a lengthy period. During the drying process of berries, microwave energy acts directly on the medium molecules (water) to transform them into heat energy. The microwave extends deep within materials being dried, allowing the interior and exterior of dried materials to be heated at the same time to prevent uneven heating and heat conduction [10]. As a result, the heating pace is rapid and unaffected by the shape of the heated object. Furthermore, strong microwave penetration causes the heated material’s interior temperature to rise quickly, considerably enhancing the drying quality and dehydrating effect. Compared to the traditional convective drying process, microwave drying drastically reduces the heating time because of the microwave’s superior thermal efficiency and extremely high penetrability [11]. Nevertheless, Hossan et al. [12] found that the shape and size characteristics of materials greatly affect the evenness of heating through microwaves. According to a study by Zheng et al. [13], the temperature changes in berries exposed to microwave energy follow a three-phase pattern: gentle, rapid, and slow rising. This pattern aligns with the sigmoidal function law.

The underlying principles of heat and mass transfer during microwave drying can be understood by examining the process of microwave energy absorption and conversion as well as creating a model for the material’s internal temperature and moisture distribution. Kowalski et al. [14], Zhou and Wang [15], and Yhang et al. [16] discovered that the way materials absorb and convert microwave energy can be precisely evaluated through changes in thermal and dielectric properties that occur with temperature and moisture. By adjusting the input of microwave power, the uniformity of the materials’ internal temperature and the outcome of the drying process can be improved. One issue with drying berries using a microwave is that the high internal temperature during the process causes a considerable decrease in the thermolabile bioactive compound. This is because the berries absorb most of the microwave energy, and very little is dissipated in the air or equipment, making the heating process highly efficient. However, the microwave also provides better control over the drying process and can eliminate microorganisms through both thermal and non-thermal means, when microwave energy disrupts the cell membranes of the microorganisms, rendering them unable to survive.

The combined drying process, namely the combination of convective and microwave dehydration, has significantly enhanced the drying effectiveness, efficiency, and energy utilization rate. Innovations in drying technology have led to the development of new and adaptable methods such as combined microwave drying. These methods improve the dehydration process by shortening drying time, increasing energy efficiency, or improving product quality. However, they may not always be cost-effective, and their feasibility varies depending on the specific material.

The study aimed to examine the dehydration behavior of chokeberries using various drying methods and evaluate their effects on drying kinetics, physical properties (such as water holding capacity), and sensory quality. In addition, the goal was to provide a detailed description of the drying behavior of chokeberries using different drying routes (microwave, microwave/convective dehydration).

## 2. Materials and Methods

### 2.1. Materials

Chokeberry fruits (black aronia, *Aronia melanocarpa* L.) were collected in August 2022 in the Paraćin area (43.88206282537799, 21.410884502500544 Decimal Degree, Serbia) and stored in the freezing chamber at a temperature of −18 °C for no longer than 4 months. The sample of each tree consisted of 15−20 randomly collected berry samples from the tree. Before each dehydration process, the fresh chokeberries were taken from the freezing chamber, treated with cold water (11–12 °C, washed for 5–10 s, and discarded damaged or unripe berries), and allowed to stabilize for a few hours at room temperature. 

### 2.2. Methods

#### 2.2.1. Microwave Dehydration 

The microwave oven (Intertronic WD900ESL25RII-2, Input 1400 W, 230 V, 50 Hz, 1200 W heater, 1400 W, convection, 900 W, output 2450 MHz) was used for the thin-layer microwave and a combination of microwave and convective drying of chokeberries. The mass load of dehydrated fruits was 96.30 kg·m^−3^ (30 g per tray). The MD was obtained at the microwave power (MW) power (P) 270, 450, and 900 W. The MCD was a discontinuous model: 2.30 (9 s of MW on 900 W, 12 s of CD on 230 °C), 2.00 (9 s of MW on 900 W, 12 s of CD on 200 °C), and 1.50 (15 s of MD on 900 W, 7 s of CD on 180 °C). The weight of the trays was measured at intervals of 5 min for MCD and 10 min for MD (in triplicates). The drying kinetic was based on mass losses of chokeberries [17,18].

#### 2.2.2. Models of Thin-Layer Dehydration

Semi-theoretical models could be used to describe the thin-layer dehydration process of fruits such as chokeberries [19]. These semi-theoretical models are described by the temperature/power of the dehydration process, relative humidity, hot airspeed, moisture content, material thickness and sphere diameter, size, and its ability to determine moisture diffusivity [20]. The water loss (moisture ratio, MR) could be reduced because equilibrium moisture content (M_e_) was typically insufficient and could be deleted without significantly changing MR Equation (1).

(1) $$\mathrm{MR}=\frac{M_{t\;}-{\;M}_{e}}{M_{o}-{\;M}_{e}}=\frac{M_{t}}{M_{0}}$$

M_t_ and M_o_, respectively, represent the moisture content reached after the convective drying time t and the initial moisture content.

The change in the total mass of fruits (M_i−1_ − M_i_) in the time between two measurements (t_i−1_ − t_i_) on a specific tray during the drying process can also be used to express the drying kinetics (drying ratio, DR, Equation (2)):

(2) $$\mathrm{DR}=\frac{M_{i-1\;}-{\;M}_{i}}{t_{i-1}-{\;t}_{i}}$$

#### 2.2.3. Determination of Effective Moisture Diffusivity

Fick’s second low-diffusion model was used to describe the moisture transport phenomena from chokeberries, and the effective moisture diffusivity (D_eff_) was determined. Equations (3) and (4) define the theoretical calculation model based on the product geometry (sphere is the appropriate model for the berries) [7,17]:

(3) $${\mathrm{MR}=A}_{1\;}\times \overset{\alpha \propto }{\underset{i=1}{\sum }}\frac{1}{J_{o}^{2}}{\;\times \;e}^{-\frac{J_{0}^{2}{\;\times \;D}_{\mathrm{eff}}}{A_{2}}}$$

(4) $$A_{1}=\frac{6}{\pi^{2}};\;A_{2}{=4\;\times \;r}^{2}$$

D_eff_ is the effective moisture diffusivity (m^2^ s^−1^), t is time (s), MR is the moisture ratio, J_0_ is the roots of the Bessel function, A_1_ (dimensionless) and A_2_ are geometric constants (mm^2^), and r is the radius of the sphere (mm).

For constant D_eff_ values and a relatively long drying period, Equation (3) is derived:

ln (MR) = ln (a) − k × t(5)

(6) $$k=-\frac{\pi^{2\;}\times {\;D}_{\mathrm{eff}}}{A_{2}}$$

The relationship between ln (MR) and t is linear (Equation (5)), and the slope is equal to the drying constant (k), making it easy to determine the constant D_eff_ (Equation (6)).

#### 2.2.4. Determination of Activation Energy

Instead of using air temperature for convective drying, the mass-to-microwave power ratio parameter was used (m × P^−1^), and the natural logarithm of D_eff_ versus mass load power^−1^ was used to determine the activation energy of MD. As a result, in the measured MD power range, the plot is a straight line, indicating the Arrhenius dependence (Equation (7), [18]).

(7) $$D_{\mathrm{eff}}{=D}_{0}{\times \;e\;}^{-{\;E}_{a\;}\times \;\frac{m}{P}}$$

E_a_ is the activation energy (W g^−1^), m is the mass load (g), D_0_ is the pre-exponential factor (m^2^ s^−1^), and P is the power of MD (W). The conversion factor between W g^−1^ and kJ mol^−1^ is 1 W g^−1^ × M (1 g mol^−1^) × 1 kJ (1000 J)^−1^.

An Arrhenius equation (Equation (8)) was used to describe the activation energy of CD:

(8) $$D_{\mathrm{eff}}{=D}_{0}{\;\times \;e}^{-\frac{E_{a}}{R\;\times \;T}}$$

E_a_ is the activation energy (kJ·mol^−1^), R is the universal gas constant (8.3143 J·mol^−1^ K^−1^), and T is the absolute air temperature (K). The previous Equation (8) could be simplified into the linear equation ln (D_eff_) = ln (D_0_) − 10^−3^ × k × (T + 273.15)^−1^. E_a_ was calculated from the slope of the Arrhenius equation (Equation (9)):

(9) $$k=-\frac{E_{a}}{R}$$

#### 2.2.5. Determination of Energy Consumption of Dehydration Processes

Determination of energy consumption (E) of both MW and MW/C was measured by using Prosto PM 001 (230 V, 50 Hz, 0–16 A, 2–3680 W, 0–9999 kWh, −10 °C to +40 °C, ≤85% of relative humidity, the altitude of use max 2000 m). The E was mathematically correlated with the CO_2_ emission during the MW and MW·C^−1^ (1 kWh of E releases 0.998 kg CO_2_ [17].

#### 2.2.6. Water-Holding Capacity

The water-holding capacity (WHC) of dehydrated chokeberries was measured to assess their hydrating abilities. By combining 10 g of powder samples with 100 mL of distilled water and letting them hydrate for 12 h (a room temperature), the WHC of dehydrated chokeberries was determined. After that, the extra water was taken out and weighed. Grams of water were used to represent the WHC about dry solids [21]. According to the AOAC method [22], dry matter in the chokeberries was determined using the gravimetric method.

#### 2.2.7. Sensory Evaluation

Detailed sensory characteristics of the fresh (unfrozen) and then frozen and dehydrated chokeberries were also evaluated. An expert team of five qualified evaluators examined fruit samples using point-based sensory analysis. Four characteristics of chokeberries—appearance, taste, scent, and consistency—were evaluated using a maximum of 5 points (on a half-point scale), with a maximum of 20 points [23].

#### 2.2.8. Statistical Methods

The statistical analysis was performed using StatSoft Statistica (ver. 12.0, Electronic Statistics Textbook, Tulsa, OK, USA). To study the differences between groups, post hoc Tukey HSD test and the second-order polynomial models Microsoft Excel (ver. 2016, Microsoft Corporation, Redmond, WA, USA) (*p* < 0.05) were used [17]. A one-way analysis of variance (ANOVA) was used to confirm the significance of variations in the quality features of the examined chokeberries. To compare and contrast all analyzed parameters of the chokeberries (WHC, D_eff_, t, E, CO_2_, and sensory properties) that were evaluated using a covariance matrix, the principal component analysis (PCA) was applied [24]. Pearson correlation was calculated, and the level of significance of *p* < 0.05 was applied. R Studio 1.4.1106 program was used for the color correlation graph between the obtained mass transfer rate parameters, the WHC, D_eff_, t, E, CO_2_, and sensory properties [25].

## 3. Results and Discussion

### 3.1. Models of Thin-Layer Dehydration

The fresh chokeberries’ initial moisture content was 3.27 ± 0.42 kg H_2_O kg^−1^ d.m. (dry matter). The MR in dehydration measures the amount of moisture present in a food product being dried using microwave and convective energy. It is an essential parameter in drying because it determines the rate at which moisture is removed from the product as well as the final moisture content of the product. A lower MR results in faster drying and lower final moisture content, while a higher MR results in slower drying and higher final moisture content [26]. The DR as well as MR in a microwave also depend on several factors, including the type and thickness of the material being dehydrated, the strength of the microwave and the temperature of convective dehydration, and the duration of the dehydration process. Providing a general dehydration rate for microwave dehydration is difficult, as it can vary significantly depending on these factors [27]. This could be attributed to the water molecules inside the berries absorbing microwave energy, causing rapid evaporation and partial puffing. In general, materials with a higher moisture content will dehydrate faster than those with a lower moisture content. Thinner materials also tend to dehydrate faster than thicker materials [14,27]. A higher-wattage microwave and a higher-temperature range of convective dehydration will generally produce a faster dehydration ratio than a lower-wattage microwave. Finally, the duration of the dehydration process will also affect the rate at which the material dehydrates [28].

**Figure 1:**
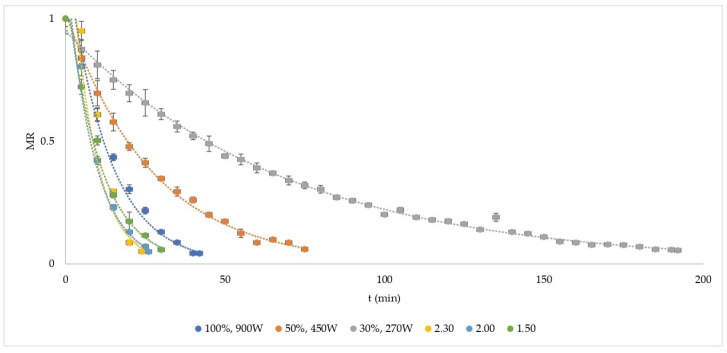
Effects of dehydration method (MD, MCD) on MR.

Lewis’s, Newton’s, Page’s, and Henderson and Pabis’s exponential models and the polynomial model are some of the most appropriate semi-theoretical models to describe the MR and DR of chokeberry dehydration, respectively [29]. Henderson and Pabis’s model for MR and the polynomial model for DR were used in this work to describe the dehydration data and discuss the phenomena. The MR can be controlled by adjusting the power of the dehydration energy, the product’s size and shape, and the drying environment’s temperature and humidity. Regardless of the drying method used, rapid water loss was noted during the initial stage of dehydration (Figure 1). For example, Henderson and Pabis model’s exponential model (MR = a × e^−k × t^) showed some mathematical regularity in the coefficient a and the coefficient k reduction, unlike slight variations in the coefficient of the polynomial model by energy growth input. In Calín-Sánchez’s research, Henderson and Pabis’s exponential models were also the appropriate model to describe the freeze and convective drying, vacuum microwave drying, and combined drying methods (convective/osmotic–vacuum microwave drying [4]).

**Figure 2:**
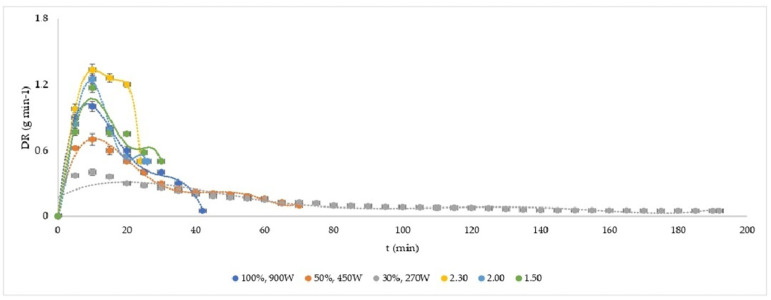
Effects of dehydration method (MD, MCD) on DR.

Both models showed high values of the coefficient of determination R^2^. The maximum DR was achieved in 6–10 min of the dehydration process regardless of the dehydration model. The highest DR had the MCD models of 1.33, 1.25, and 1.17 g min^−1^ for the models 2.30, 2.00, and 1.50, respectively. Conversely, the highest DRs for the MD models were 1.04, 0.72, and 0.39 g min^−1^ for the microwave power ranges 900, 450, and 270 W, respectively (Figure 2).

All curves for drying kinetics had the same shape, with different drying times to a constant mass. Drying time t (Table 1) statistically significantly depended directly on the chosen dehydration method.

The MCD method, which implies the MW power of 900 W for 9 s and the CD temperature for 12 s, proved to be the most effective without statistical significance (the shortest t, 24 ± 2 min). The shortest MD time was a 900 W model (42 ± 4 min), which is consistent with the results of the vacuum–microwave drying in Calín-Sánchez’s work (54 min, [4]). When the microwave power was reduced, temperature curves were separated, leading to noticeably longer MD durations of up to 192 min.

Because of the MD’s distinctive heating, a significant vapor pressure difference developed between the center and the surface of the chokeberry sphere shape. Because water evaporation from the thin surface was limited, the berries’ rapid surface hardening occurred rapidly, affecting the drying process’s extension. The MCD method, regardless of the energy range input, showed a slight variation in t.

### 3.2. Determination of Effective Moisture Diffusivity

The D_eff_, also known as the effective moisture diffusivity, is a measure of the rate at which moisture diffuses through a material. It is affected by several factors, including the properties of the material, the temperature and humidity of the environment, and the presence of any barriers or coatings on the material. In general, the effective moisture diffusion coefficient will be higher at higher temperatures and energy inputs, as the increased temperature will cause the moisture molecules to move more quickly [5]. It is not clear how microwave energy alone would affect the effective moisture diffusion coefficient, as it is not a property of the material. However, if the material is heated by microwave energy, the effective moisture diffusion coefficient will be higher at higher temperatures [18]. The experimental results in this paper confirmed this claim, where the highest D_eff_ values had the MCD models with the highest microwave power range 900 W for 9 s and the CD temperature for 12 s (D_eff_ = 6.0768 × 10^−9^ ± 5.9815 × 10^−11^ m^2^ s^−1^). When increasing the MW energy input or being exposed to microwave energy for a long time, the D_eff_ will statistically significantly increase.

### 3.3. Determination of Activation Energy

The effect of microwave and convective dehydration on the E_a_ may depend on the specific reaction and conditions. However, it is known that microwave heating can cause localized heating and can lead to the formation of hot spots, which can potentially increase the reaction rate by increasing the temperature and the collision frequency of molecules [30]. The Ea value for the MD was 81.6231 ± 0.0787 kJ·mol^−1^ and for the MCD was 92.8707 ± 0.0942 kJ·mol^−1^. Decreased E_a_ indicates more effective moisture diffusivity (higher D_eff_) and rising moisture diffusion with sphere radius (thickness), implying that lower energy consumption causes the bond between the water molecules of the sample to break [31].

### 3.4. Determination of Energy Consumption of Dehydration Processes

Assessing the energy consumption of dehydration processes can help determine the efficiency of the process and identify opportunities for energy savings. One way to approach this assessment is direct measurement; the energy consumption analysis of the dehydration process was to measure the energy input to the system directly. The experimental results (Table 1) showed that the dehydration process has a strong impact on energy consumption (E) and was directly related to the duration of the drying process. It was apparent that the energy input statistically significantly (*p* < 0.05) decreased with the drying MW energy increase and subsequent drying time decrease. The MCD was less energetically demanding for the chokeberry dehydration, especially the model 2.30 (E_min_ = 0.382 ± 0.036 kWh). In previous research [32], the higher temperature of chokeberry convective dehydration (70 °C) indicated a more energy-efficient process (2474.35 ± 15.74 kJ, 0.6873 ± 0.01 kWh).

### 3.5. Water-Holding Capacity

**Figure 3:**
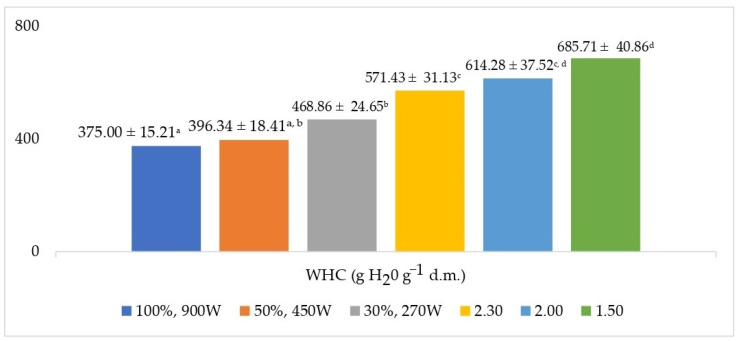
WHC in chokeberry obtained by two methods: MD and MCD. ^a–d^ Different letters in the superscript in Figure 3 indicate a statistically significant difference between values at a significance level of *p* < 0.05 the post hoc Tukey HSD test).

WHC refers to the amount of water that a dehydrated material can retain. In the context of fruit dehydration, the water-holding capacity of the fruit will affect how long it takes to dehydrate and how much the fruit shrinks during the dehydration process. Fruits with a high WHC, such as melons and citrus fruits, will take longer to dehydrate and may shrink less during the process than fruits with a low WHC, such as berries and grapes. This is because the high-WHC fruits have a higher moisture content and are more challenging to dry. The choice of the dehydration method as well as the input energy has a statistically significant effect on the WHC results (Figure 3). A higher WHC characterized the chokeberries obtained by the MCD method compared to the MD method. The results showed that at the same microwave energy and with prolonged effect, lower temperature, and shorter convective dehydration time, dried chokeberries will have maximum WHC (685.71 ± 40.86 g H_2_O g^−1^ d.m.) and less shrinkage. The previous results [33] showed a higher WHC obtained by convective dehydration (777.21–924.037 g H_2_O g^−1^ d.m.). Different methods such as fluidized-bed jet milling and drying (FJMD) or freeze drying (FD) will provide the following WHC of chokeberries: 661 ± 19 g H_2_O g^−1^ d.m. and 812 ± 20 g H_2_O g^−1^ d.m., respectively [34].

The WHC of fruit could be affected by factors such as the type of fruit, the stage of ripeness, and the storage conditions. For example, overripe fruit may have a higher WHC than ripe but not yet overripe fruit. Similarly, fruit that was stored in a humid environment may have a higher WHC than fruit stored in a dry environment.

### 3.6. Sensory Evaluation

**Figure 4:**
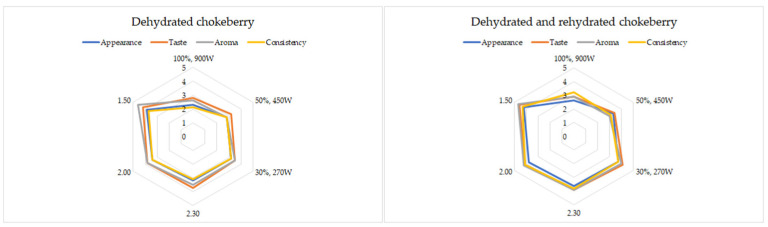
Sensory evaluation of dehydrated and dehydrated/rehydrated chokeberries.

Sensory evaluation of dehydrated involves assessing the appearance, taste, aroma, consistency (texture), and total score of the dried fruits to determine their quality and overall acceptability [35]. A group of five food industry engineers and professors with experience and knowledge of the sensory evaluation approach completed the assessment in a laboratory environment. Factors that are commonly evaluated include the color, texture, and amount of moisture in the fruit as well as any off-flavors or odors that may be present. The evaluation results can be used to make adjustments to the drying process or to the storage conditions of the dehydrated fruits to improve their quality and extend their shelf life. The choice of the dehydration method has a statistically significant (*p* < 0.05) effect on the results of the sensory evaluation (Figure 4). The MDs (100%, 900 W and 50%, 450 W) were very extreme for chokeberry dehydration, and the dehydrated berries were almost burned, with a low index of appearance (the higher shrinkage, MD 900 W 2.30 ± 0.27 and MD 450 W 2.30 2.60 ± 0.22) and consistency (MD 900 W 2.10 ± 0.22 and MD 450 W 2.80 ± 0.27). As a general comment, it should be mentioned that the burnt flavor could be avoided by lower MD energy input.

The MCD 1.50 model after rehydration had the greatest evaluations for sensory attributes in terms of all properties; notably, the total score (17.80 ± 0.76), the aroma (flavor, 4.70 ± 0.27), and the taste, which was particularly highlighted, were comparable to fresh fruit in rehydrated chokeberry. The sensory attribute of aroma is the most important and best-rated in MCD dehydration and the taste in MD dehydration. MCD dried berries, compared to MD berries, were characterized by greater freshness, acidity, and astringency, all similar to fresh chokeberry. The use of combined microwave and convective drying resulted in the greater freshness of dried and rehydrated chokeberry but not in greater intensity of acidity, bitterness, and astringency. A higher temperature of convective drying and a higher microwave power affected the slightly increased crispness of dried chokeberry fruits regarding their texture. Similar results were found in the dehydration of other berries such as blueberries [36].

### 3.7. Statistical Methods

**Figure 5:**
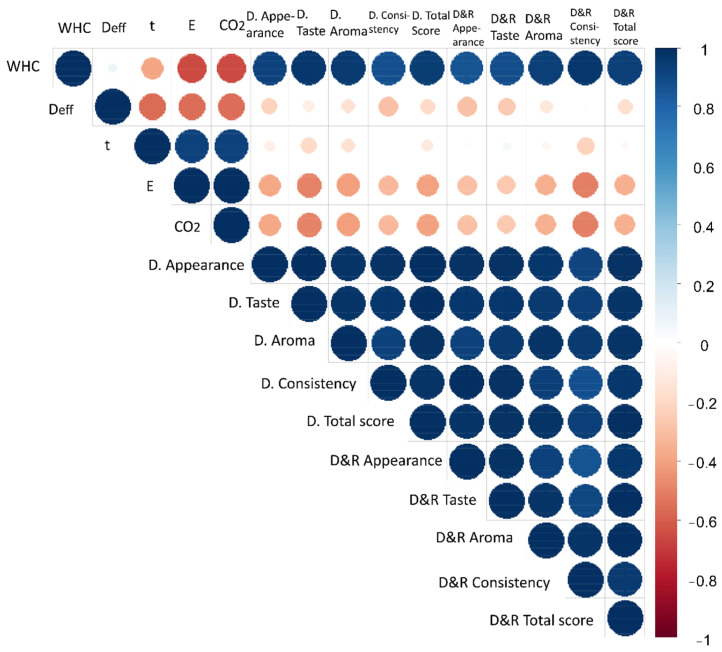
Color correlation diagram between the parameters of the independent variables and the responses of dehydration method.

The color correlation analysis investigated the connections between observed chokeberry samples (Figure 5).

A color correlation diagram was created to show the statistical significance of the correlation coefficients between the different variables and the responses [37]. The size of the circles and the color (blue for positive correlation, red for negative correlation) are used to graphically display the values of the correlation coefficients between the tested parameters (Figure 5). A high level of positive correlation was shown between most of the responses of the dehydration methods, while there was a high level of negative correlation between the dependent variable WHC and E and CO_2_ (r = −0.6645 and r = −0.6637, respectively, statistically significant at *p* < 0.05). A lower level of negative correlation was found between the D_eff_ and t (r = −0.5609), E (r = −0.5464), and CO_2_ (r = −0.5458). Furthermore, a negative correlation between E and CO_2_ and some sensory characteristics (such as consistency and taste) was also noticed. The highest positive correlations were noticed for E and CO_2_, r = 0.9999 statistically significant at *p* < 0.05). From Figure 5, the highest positive correlations were also observed for WHD and D. appearance (r = 0.9275), D. taste (r = 0.9624), D. aroma (r = 0.9507), D. total score (r = 0.9423), D&R aroma (r = 0.9373), and D&R consistence (r = 0.9696), D&R total score (r = 0.9344), statistically significant at *p* < 0.05). There is as well a high positive correlation between t and E and CO_2_ (r = 0.9158 and r = 0.9161, respectively, statistically significant at *p* < 0.05). In addition, a high positive correlation was noticed between all sensory characteristics, r, ranging from 0.9182 to 0.9988.

**Figure 6:**
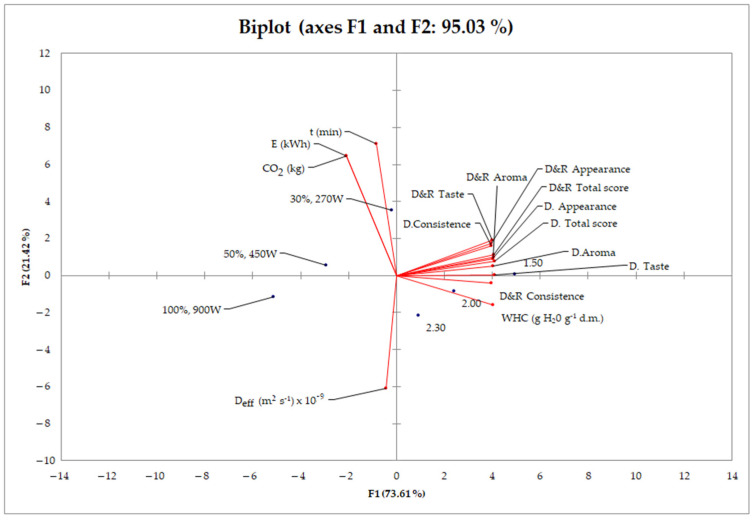
PCA of independent variables and responses the WHC, D_eff_, t, E, CO_2_, and sensory properties. The red lines are the original variables in the space of the first and second components.

The type of dehydration method and its parameters were used as independent variables, and PCA was applied to identify the structure in the correlation between these parameters and dependent variables, the WHC, D_eff_, t, E, CO_2_, and sensory properties (Figure 6). The angles between corresponding variables indicate the degree of their correlations, where small angles correspond to high correlations [38]. A scatter plot was created with the first two principal components (PC1, PC2) from the PCA data matrix. The first two PCs demonstrated 95.03% of the total variance in the laboratory data. This method allows for visualization of the trends in the displayed data and shows the discriminating efficiency of the used descriptors. The contribution of the variables (%) showed that WHC (8.5990%) and each of dehydrated and dehydrated/rehydrated sensory characteristics (appearance, taste, aroma, consistency, and total score with average value 8.3647–8.9748%) most participated in PC1 and D_eff_ (19.6556%), t (27.1077%), E (22.3598%), and CO_2_ (22.3643%) in PC2. The parting within samples could be seen from the PCA figure, where there is a clear separation of samples according to the dehydration method. Therefore, the position of the samples in Figure 6 was primarily more influenced by the type of dehydration method (MCD, MD) than the parameters of the dehydration method.

The MCD methods (1.50, 2.00, and 2.30) were characterized by higher values of analyzed parameters WHC and sensory properties of dehydrated and dehydrated/rehydrated chokeberries (oriented on the positive side of the x-axis by the positive value of the PC1 component) compared to the MD methods oriented on the negative side of the x-axis (by the negative value of the PC1 component) and characterized by D_eff_, t, E, and CO_2_. Therefore, the MCD method (1.50) was characterized by the high values of the following response: sensory properties of dehydrated and dehydrated/rehydrated chokeberries except for the consistency. The MCD methods (2.00 and 2.30) were characterized by the high values of the following responses: WHC and consistency.

The key findings according to Henderson and Pabis for MR and polynomial for DR are that rapid water loss occurs during the initial stage of dehydration. The maximum DR was achieved in 6–10 min of the dehydration process regardless of the dehydration model used. The MCD method proved to be the most effective in terms of drying time, with a statistically significant shorter time of drying process. The discussion also notes that the distinctive heating associated with the MD method can lead to a significant vapor pressure difference between the center and surface of the chokeberry sphere shape, resulting in rapid surface hardening, which can affect the drying process’s extension. This effect was not observed in the MCD method, which showed a slight variation in drying time regardless of the energy range input.

The effective moisture diffusion coefficient typically increases with increasing temperature and energy input. The reason for this is that higher temperatures increase the movement of moisture molecules, resulting in a faster diffusion rate. The experimental results presented in the paper support this statement, indicating that the D_eff_ values were highest for the MCD models with the highest microwave power range. As the energy input increases, the D_eff_ also increases significantly. Lowering the E_a_ indicates more effective moisture diffusivity (higher D_eff_), and increasing the moisture diffusion with sphere radius (thickness) suggests that lower energy consumption causes the bond between the water molecules of the sample to break. The experimental results discussed in the paper support the notion that the D_eff_ values are significantly affected by the energy input, with higher values obtained for higher microwave power ranges. Understanding the factors that affect the D_eff_ is critical for optimizing the moisture removal process in materials, particularly for industrial applications where efficient moisture removal is essential.

The experimental results mentioned in the discussion demonstrate that energy consumption is strongly affected by the duration of the drying process. Specifically, the results showed that the energy input statistically significantly decreased with the increase in MW energy and subsequent decrease in drying time. This indicates that reducing the duration of the drying process can lead to significant energy savings. It is important to note that different dehydration processes may have varying energy demands. The discussion also highlights that MCD was less energetically demanding for the chokeberry dehydration process. This suggests that choosing the appropriate dehydration method for a specific product can also help to reduce energy consumption.

The choice of dehydration method and input energy can significantly impact the WHC results. For instance, the WHC results obtained by using the MCD method were higher compared to those obtained by the MD method for chokeberries. This could be because the MCD method combines the effects of both convective and microwave energy, which helps to increase the diffusion of moisture and promotes faster dehydration, resulting in increased WHC. It is also important to note that the drying conditions can also affect the WHC results. For example, in the case of chokeberries, the maximum WHC and less shrinkage were obtained when using a lower temperature, shorter convective dehydration time, and prolonged microwave energy exposure. This could be because the lower temperature and shorter dehydration time help to reduce the diffusion of moisture and, combined with prolonged microwave energy exposure, promote better water evaporation, leading to higher WHC and less shrinkage.

The choice of dehydration method can significantly affect the results of sensory evaluation, and the degree of moisture removal can also impact the quality of the final product. In the case of chokeberry dehydration, for example, excessive MD resulted in burnt flavors and a low index of appearance and consistency. MCD is one dehydration method that has been shown to produce high-quality dehydrated fruits with excellent sensory attributes. Compared to other methods, MCD resulted in greater freshness, acidity, and astringency, all similar to fresh chokeberry. The taste of rehydrated MCD-dried berries was found to be comparable with fresh fruit, which is a significant advantage. A higher temperature of convective drying and higher microwave power of MCD can slightly increase the crispness of dried chokeberry fruits, improving their texture.

The findings have implications for optimizing the drying process, especially in the food industry, where dehydration is an important step in fruit processing and preservation.

## 4. Conclusions

According to the presented results, it can be concluded that a rapid water loss was observed during the initial stage of dehydration regardless of the drying method used. Both models showed high values of the coefficient of determination R^2^. Drying time was found to be directly dependent on the chosen dehydration method, with the MW power of 900 W for 9 s and the CD temperature for 12 s being the most effective. The dehydration process has a strong impact on energy consumption and was directly related to the duration of the drying process. The MD was less energetically demanding for the chokeberry dehydration, especially the model 2.30. The highest D_eff_ values had the MD models with the highest microwave power range. A higher WHC characterized the chokeberries obtained by the MCD method compared to the MD method. At the same microwave energy, with prolonged effect, lower temperature, and shorter convective dehydration time, dried chokeberries will have maximum WHC and less shrinkage. MCD dried berries compared to MD berries were characterized by greater freshness, acidity, and astringency, all similar to fresh chokeberry, and affected the slightly increased crispness of dried chokeberry fruits regarding their texture. The study provides valuable information for developing new and efficient drying methods for chokeberries.

## Figures and Tables

**Table 1:** Average values and standard deviations of t, D_eff_, E, CO_2_, and experimental models for MR and DR of MD and MCD of chokeberry.

Model	T (min)	D_eff_ (m^2^ s^−1^)	Henderson and Pabis Model	Polynomial Model	E (kWh)	CO_2_ (kg)
100%, 900 W	42 ± 4 ^a^	4.4891 × 10^−10^ ± 4.4518 × 10^−12^, ^f^	y = 1.1181 × e^−0.0730 × x^ R^2^ = 0.9881 MSE = 0.0025	y = 2.5657 × 10^−7^ × x^5^ − 3. 3384 × 10^−5^ × x^4^ + 1.6315 × 10^−3^ × x^3^ − 3.6273 × 10^−2^ × x^2^ + 0.3231 × x + 0.0850 R^2^ = 0.9706 MSE = 0.0165	0.624 ± 0.059 ^b^	0.623 ± 0.059 ^b^
50%, 450 W	75 ± 6 ^b^	1.7492 × 10^−10^ ± 2.0241 × 10^−12^, ^e^	y = 1.0134 × e^−0.0366 × x^ R^2^ = 0.9999 MSE = 0.0002	y = 1.5705 × 10^−8^ × x^5^ − 3.6291 × 10^−4^ × x^4^ + 3.0319 × 10^−3^ × x^3^ − 1.0989 × 10^−2^ × x^2^ + 0,1513 × x + 0.0029 R^2^ = 0.9950 MSE = 0.0059	0.640 ± 0.062 ^b^	0.638 ± 0.062 ^b^
30%, 270 W	192 ± 15 ^c^	3.6891 × 10^−9^ ± 3.3214 × 10^−11^, ^a^	y = 0.9432 × e^−0.0147 × x^ R^2^ = 0.9979 MSE = 0.0001	y = 3.4667 × 10^−10^ × x^5^ − 1.5067 × 10^−7^ × x^4^ + 2.3610 × 10^−5^ × x^3^ − 1.5861 × 10^−3^ × x^2^ + 0.0394 × x + 0.0393 R^2^ = 0.848 MSE = 0.0069	0.866 ± 0.081 ^c^	0.865 ± 0.081 ^c^
2.30	24 ± 2 ^a^	6.0768 × 10^−9^ ± 5.9815 × 10^−11^, ^b^	y = 1.3174 × e^−0.1795 × x^ R^2^ = 0.9338 MSE = 0.0344	y = 1.8931 × 10^−5^ × x^5^ − 1.0469 × 10^−3^ × x^4^ + 0,0186 × x^3^ − 0,1157 × x^2^ + 0,2901 × x + 3.2426 × 10^−10^ R^2^ = 0.9999 MSE = 0.0893	0.382 ± 0.036 ^a^	0.381 ± 0.357 ^a^
2.00	26 ± 2 ^a^	1.5412 × 10^−9^ ± 1.5374 × 10^−11^, ^d^	y = 1.2133 × e^−0.1167 × x^ R^2^ = 0.9560 MSE = 0.005	y = 1.1634 × 10^−6^ × x^5^ + 9 × 10^−5^ × x^4^ − 1.8603 × 10^−3^ × x^3^ + 0.0042 × x^2^ + 0.1998 × x − 0.0030 R^2^ = 0.9970 MSE = 0.1205	0.396 ± 0.036 ^a^	0.395 ± 0.363 ^a^
1.50	30 ± 3 ^a^	1.3565 × 10^−9^ ± 1.2355 × 10^−11^, ^c^	y = 1.1058 × e^−0.0948 × x^ R^2^ = 0.9872 MSE = 0.0014	y = 1.3537 × 10^−6^ × x^5^ − 1.2626 × 10^−3^ × x^4^ + 0.0044 × x^3^ − 0.0709 × x^2^ + 0.4884 × x − 0.0010 R^2^ = 0.9917 MSE = 0.6254	0.451 ± 0.048 ^a^	0.450 ± 0.447 ^a^

^a–f^ Different letters in the superscript in Table 1 indicate a statistically significant difference between values at a significance level of *p* < 0.05 (the post hoc Tukey HSD test).

## Data Availability

Data are contained within the article.
